# Acute effects of the coffee diterpene cafestol on glucose metabolism in non-diabetic subjects with abdominal obesity

**DOI:** 10.1900/RDS.2023.19.34

**Published:** 2023-06-30

**Authors:** Fredrik Drews Mellbye, Kjeld Hermansen, Per Bendix Jeppesen, Søren Gregersen

**Affiliations:** 1 Steno Diabetes Center Aarhus, Aarhus University Hospital, Aarhus, Denmark; 2 Department of Endocrinology and Metabolism, Aarhus University Hospital, Aarhus, Denmark; 3 Department of Clinical Medicine, Aarhus University, Aarhus, Denmark.

**Keywords:** cafestol, coffee, type 2 diabetes mellitus, insulin resistance, obesity, humans, randomized controlled trial, cross-over studies

## Abstract

**Objectives:**

Coffee consumption is associated with a reduced risk of Type-2 Diabetes. A bioactive compound in coffee, cafestol, has shown potential preventive effects for Type-2 Diabetes in cell and animal studies, but its potential benefits in humans have not been examined.

**Methods:**

In this randomized, double- blinded crossover intervention study, 15 healthy participants with increased waist circumference and thus elevated risk of developing Type-2 Diabetes underwent three oral glucose tolerance tests one week apart, with placebo, 7 mg- or 14 mg cafestol capsules ingested with the glucose load.

**Results:**

There were no substantial differences in area under the curve (AUC) for glucose, insulin, glucagon-like peptide 1 (GLP-1) or gastric inhibitory peptide (GIP) on placebo or cafestol intervention study days. Among participants with impaired glucose tolerance and/or elevated fasting glucose (n=8, 53%), ingestion of 14 mg of cafestol resulted in an 11% larger AUC for GIP (p=0.046) and a 5% smaller AUC for glucose (p=0.14), compared to placebo.

**Conclusions:**

Our results suggest that cafestol may contribute to coffee's inverse association with risk of Type-2 Diabetes, particularly in subjects with impaired glucose tolerance, possibly through increased GIP secretion. Further studies are needed to confirm these novel findings in participants with impaired glucose metabolism, both after acute and longer-term cafestol intervention.

## 1 Introduction

Type-2-diabetes [T2D] is a growing health concern, affecting one in eleven adults worldwide. T2D can lead to severe complications including blindness, kidney failure, heart attack, stroke and amputations if insufficiently treated [1]. Lifestyle changes, such as diet and exercise, can help prevent or delay disease onset [2]. Unfortunately, adhesion to lifestyle changes is cumbersome and with low adherence. Modern antidiabetic drugs have made great health improvements for T2D patients; however, the most recent pharmaceutical innovations are expensive and financially inaccessible for vast parts of the world’s populations of T2D subjects. Therefore, novel inexpensive means of preventing or treating T2D are needed. Coffee consumption has potential as a preventive measure. According to large epidemiological studies, dose-dependent inverse relationships exist between coffee consumption and development of T2D [3, 4]. Daily coffee consumption of four cups is associated with a 30% lower relative risk of developing T2D compared to consumption of zero or one cup of coffee every day [3]. Coffee contains many bioactive substances and the specific substance[s] being responsible for its assumed beneficial effects as preventive antidiabetic substance remains unclear; nor has a definite mechanism of action been identified. So far, only few prospective interventional trials with coffee have been carried out and they have found little or no causal evidence to support the inverse associations between coffee consumption and T2D development. Most interventional coffee studies have been conducted short-time and/or using freeze-dried instant coffee [5- 7], a brew from which lipophilic substances such as cafestol and kahweol have been removed.

In this context it is noteworthy that cafestol has shown promising beneficial effects on glucose metabolism in cell experiments and animal intervention studies [8,9]. In 2015, we discovered that cafestol increases glucose uptake in a human muscle cell line and potentiate glucose-stimulated insulin secretion from clonal rat beta cell, INS-1E cells [8]. Subsequently, we found that a 10-week treatment period with cafestol lowered blood glucose levels, lowered glucagon levels, improved insulin sensitivity and induced a higher insulin secretory capacity *in vitro* from isolated islets of Langerhans [9] in the insulin resistant and obese KKAy mouse model [10].

It remains to be examined if cafestol contributes to coffee’s apparent diabetes preventive effects in humans. Whereas caffeine-free coffee has been thoroughly investigated, no cafestol interventional study has so far been performed in humans with endpoints concerning T2D development or T2D-related surrogate markers. The aim of this study was to examine acute effects of low [7 mg] and high [14 mg] cafestol dosages on glucose metabolism in healthy subjects during an oral glucose tolerance test [OGTT] in a randomized, double-blinded, placebo-controlled crossover study. We hypothesized that cafestol reduces the glycemic responses during an OGTT in healthy, non-diabetic subjects with increased waist circumference.

## 2 Materials and Methods

### 2.1 Study design

This study was an acute, randomized, double- blinded placebo-controlled crossover study in participants at risk of developing T2D due to abdominal obesity with increased waist circumference. The participants underwent three study visits for OGTTs with approximately one-week washout period between visits.

During the two days prior to their study days, participants were not allowed to drink coffee, tea or alcohol, nor were they allowed to conduct strenuous exercise. Participants arrived at the research site at 7AM, fasting since midnight. A peripheral venous catheter was placed retrogradely into the median cubital vein in the antecubital fossa. Blood samples were drawn from the venous catheter at time points -15, 0, 15, 30, 60, 90, 120 and 180 min. At time point 0 min, the participants drank 75 g glucose dissolved in 225 ml water and were administered an intervention capsule or a placebo capsule. After three study days, all participants had received both intervention capsules [7 mg cafestol and 14 mg cafestol] and a placebo capsule.

### 2.2 Participants

The participants were recruited from responders to online advertisements such as hospital social media posts and announcements on participant-recruiting webpages. The inclusion criteria were age between 18 and 80 years and waist circumference > 102 cm for male participants or > 88 cm for female participants, based on WHO criteria for increased metabolic risk [11]. Responders with one or more of the following conditions were excluded from the study; a medical history of diabetes, HbA1c > 48 mmol/l, current or previous treatment with antidiabetic drug[s] and significant disease/disability. The study was conducted at Steno Diabetes Center Aarhus at Aarhus University Hospital, Denmark.

### 2.3 Interventions

Intervention capsules were produced by Kaffe Bueno Aps, Søborg, Denmark, who purified spent coffee grounds into a compound of 86% cafestol and 14% kahweol. The compound was dissolved in sunflower oil and sealed in starch capsules. The high-, low- and placebo-dose capsules were filled with 14 mg, 7 mg and 0 mg of cafestol-rich compound, respectively. The placebo capsules contained sunflower oil only.

### 2.4 Materials

Blood samples were drawn into tubes coated with dipotassium ethylenediaminetetraacetic acid [K2EDTA] [Becton Dickinson, New Jersey, U.S.A.] and placed on ice until centrifuged for 10 minutes with a 2985 relative centrifugal force [RCF] at 4°C before the supernatant was transferred to tubes and frozen at -80°C. All samples were batch-analysed at study end. Glucose was analysed using Roche Diagnostics COBAS C 111 analyzer [Roche Diagnostics International AG, Rotkreuz, Switzerland]. Insulin was analysed using Mercodia Insulin ELISA plates [Mercodia, Uppsala, Sweden] and PerkinElmer Victor 3 1420 Multilabel counter [PerkinElmer, MA, U.S.]. Glucagon-like peptide 1 [GLP-1] and Gastric Inhibitory Peptide [GIP] was analysed using Mercodia ELISA plates [Mercodia, Uppsala, Sweden] and PerkinElmer Envision Multimode Plate Reader [PerkinElmer, Massachusetts, U.S.A.].

### 2.5 Outcomes

Primary outcome was change in area under the curve [AUC] for glucose during the OGTT. Secondary outcomes were changes in AUC for insulin, GLP-1 and GIP.

### 2.6 Randomization and blinding

The sequence determining which study day a participant would receive a specific capsule was randomized and double blinded to participants and the investigator. The randomized sequence was generated by a trusted third-party throwing virtual dice assigning participants to color-coded capsules. Color code and capsule combination sheets were securely kept from the investigator. Capsules were administered by study laboratory technicians. The participants’ color sequences were revealed to the investigator for statistical analysis after all participants had completed all study days; however, correct capsule- color combination was not revealed to the investigator until all statistical analysis was finalized.

### 2.7 Statistical methods

All statistical analysis was performed in R using R-Studio [RStudio Team [2020]. RStudio: Integrated Development for R. RStudio, PBC, Boston, MA. URL http://www.rstudio.com/.] One-and-a-half-hour area under the curve [AUC] [1.5-hour AUC] and three- hour AUCs [3-hour AUCs] were calculated for glucose, insulin, GLP-1 and GIP curves. Every AUC was calculated individually for each intervention at every visit. AUCs were compared using repeated measures analysis of variance (ANOVA). Effect of baseline characteristics on AUCs were evaluated individually using a linear regression model [with the call: formula = AUC ~ intervention + baseline_variable]. A power calculation was performed prior to the study, suggesting a sample size of 14 participants. A total number of 15 participants was decided to accommodate one potential dropout.

## 3 Results

### 3.1 Participants and recruitment

Forty-one individuals responded to our advertisements and received informational materials. After reading the materials, 16 responders expressed interest in participating and underwent interviews and screening. One participant was excluded for not meeting the waist circumference criterion. The remaining 15 participants provided written consent after sufficient time for consideration and completed all three study days without any dropouts. Baseline data were collected during the first visit [Table 1].

**Table 1.** Baseline data

Sex	Female (n=4)	Male (n=11)	Total (n=15)
**Age (years)**			
Mean	58.2	69.5	66.5
Range	20.0 - 74.0	54.0 - 76.0	20.0 - 76.0
SD	25.6	7.4	14.3
**Weight (kg)**			
Mean	86.5	100.5	96.8
Range	82.0 - 95.0	90.0 - 114.0	82.0 - 114.0
SD	6.1	7.6	9.5
**Height (cm)**			
Mean	166.2	181.3	177.3
Range	165.0 - 169.0	171.0 - 192.0	165.0 - 192.0
SD	1.9	5.9	8.5
**BMI - (kg/m2)**			
Mean	31.3	30.6	30.8
Range	29.8 - 34.9	27.2 - 33.8	27.2 - 34.9
SD	2.4	2.2	2.2
**Waist circumference (cm)**			
Mean	102.2	110.9	108.6
Range	92.0 - 112.0	103.0 - 116.0	92.0 - 116.0
SD	9.0	4.9	7.1
**HbA1c (mmol/mol)**			
Mean	38.0	38.7	38.5
Range	35.0 - 40.0	35.0 - 47.0	35.0 - 47.0
SD	2.2	3.6	3.2
**HOMA-IR**			
Mean	12.9	19.8	18.0
Range	8.2 - 18.8	9.1 - 45.8	8.2 - 45.8
SD	5.4	13.0	11.7
**Family history of T2D**			
Yes	1 (25%)	5 (45%)	6 (40%)
**Minutes of workout pr. week**			
0	1 (25%)	1 (9%)	2 (13%)
1-119 (0-2 hours)	0 (0%)	4 (36%)	4 (27%)
120-239 (2-4 hours)	2 (50%)	4 (36%)	6 (40%)
240-479 (4-8 hours)	1 (25%)	1 (9%)	2 (13%)
480 or more (8 hours or more)	0 (0%)	1 (9%)	1 (7%)

HOMA-IR (Homeostatic Model Assessment for Insulin Resistance)

### 3.2 Outcomes

**Figure 1.**
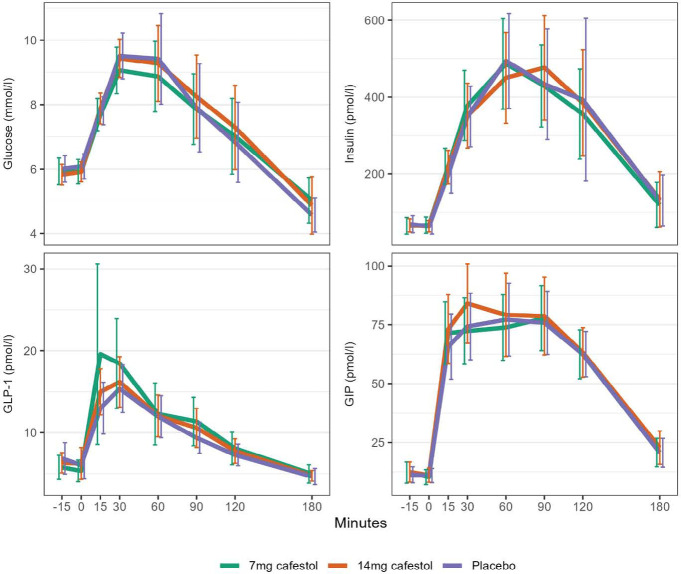
Mean curves for the impact of ingestion of 7 mg cafestol, 14 mg cafestol or placebo at time point 0 minutes on glucose, insulin, GLP-1 and GIP plasma concentrations during an oral 75 g glucose tolerance test in 15 non-diabetic subjects with abdominal obesity. Error bars represent 95% CI.

A two-way repeated measures ANOVA was performed to evaluate the effect of cafestol and placebo capsules on glucose, insulin, GLP-1 and GIP levels at each time point during the OGTT [Figure 1]. There were no significant effects of the different capsules on glucose, insulin, GLP-1 or GIP levels at any time point. The concentrations measured were mostly normally distributed in each group at each time point, as assessed by Shapiro-Wilk’s test. QQ plots also visually confirmed our assumption of normality. Results were log-transformed, but this did not alter significance levels of the repeated measures ANOVA. There were few extreme outliers. An additional repeated measures ANOVA performed only on participants without extreme outliers did not change the results; there were still no significant effects of any cafestol dose on glucose, insulin, GLP-1 or GIP levels at any time point.

Area under the curve for glucose, insulin, GLP-1, and GIP during the OGTT were individually calculated for each participant at each study day for both the 0-90 min [1.5-hour] and 0-180 min [3-hour] time intervals. The mean AUCs were subsequently then calculated for each study day and compared using repeated measures ANOVA. There was no effect of study day on blood sample levels; 7 mg- or 14 mg-cafestol capsules did not alter 1.5- or 3-hour AUCs for glucose, insulin, GLP-1 or GIP compared to placebo capsules [Table 2].

**Table 2.** 1.5- and 3-hour area under the curves for glucose, insulin, GLP-1 and GIP

	Mean	SD	vs. placebo Δ-mean	T.test vs. placebo 95% CI	T.test vs. placebo p-value
**Glucose (mmol/l x min)**					
**1.5-hour AUC**					
Placebo	778	165			
7 mg cafestol	748	135	-30	-82, 21	0.22
14 mg cafestol	777	136	-1	-61, 59	0.97
**3-hour AUC**					
Placebo	1341	318			
7 mg cafestol	1332	261	-9	-118, 100	0.87
14 mg cafestol	1375	311	34	-93, 161	0.58
**Insulin (pmol/l x min)**					
**1.5-hour AUC**					
Placebo	32615	14426			
7 mg cafestol	33299	13750	685	-4690, 6059	0.79
14 mg cafestol	32275	13684	-340	-4752, 4073	0.87
**3-hour AUC**					
Placebo	60699	37696			
7 mg cafestol	59274	27517	-1425	-15064, 12213	0.83
14 mg cafestol	60723	29565	25	-10614, 10663	0.999
**GLP-1 (pmol/l x min)**					
**1.5-hour AUC**					
Placebo	1085	405			
7 mg cafestol	1287	726	202	-214, 619	0.32
14 mg cafestol	1155	373	70	-112, 254	0.42
**3-hour AUC** Placebo	1692	585			
7 mg cafestol	1969	971	277	-269, 823	0.30
14 mg cafestol	1805	564	113	-162, 388	0.39
GIP (pmol/l x min)					
**1.5-hour AUC** Placebo	6192	2273			
7 mg cafestol	6159	2106	-33	-727, 662	0.92
14 mg cafestol	6628	2571	436	-273, 1144	0.21
**3-hour AUC** Placebo	10763	3019			
7 mg cafestol	10759	3120	-4	-912, 904	0.99
14 mg cafestol	11338	3623	575	-442, 1594	0.25

The calculated mean differences were not statistically significant. The 1.5-hour AUCs for glucose and insulin were normally distributed and had no extreme outliers, while the 3-hour AUC for glucose was not normally distributed on 7 mg and 14 mg cafestol, and 3-hour AUC for insulin was not normally distributed on placebo day. Both the 1.5- and 3-hour AUCs for GLP-1 had one extreme outlier and a not normally distributed 7 mg cafestol-group. The 3-hour AUC for GLP-1 placebo group was also not normally distributed. The 1.5-hour and 3-hour AUCs for GIP had one and two extreme outliers, respectively, and a not normally distributed 14 mg cafestol group. An additional repeated measures ANOVA test was performed, excluding outliers, but there were still no effect of study day/capsule on AUCs for glucose, insulin, GLP-1 or GIP. Log transforming the data ensured normally distributed AUCs in all groups and removal of several outliers but did not alter the ANOVA-evaluation of no effect of capsules.

### 3.3 Ancillary analyses

Ancillary analyses were conducted to investigate the effects of several variables on 1.5-hour area under the glucose-, insulin-, GLP-1- and GIP-curves, including study day [7 mg cafestol /14 mg cafestol vs placebo], HbA1c [mmol/mol], BMI [kg/m2], age [years], sex [female/male], workout [0, 0-2, 2-4, 4-8, 8+ hours pr. week], family history of diabetes [no/yes], habitual intake of cups of coffee with cafestol daily, and cups of coffee without cafestol daily.

1.5-hour AUC for glucose was found to be increased with higher HbA1c [β = 20.9, p = 0.002], BMI [β = 22.8, p = 0.03], age [β = 4.0, p = 0.009], and male sex [β = 105, p = 0.03]. 1.5-hour AUC for insulin was found to be increased with BMI [β = 2610, p = 0.007] and reduced with workout [β = -5242, p = 0.006]. There was no significant effect of any variable on 1.5-hour AUC for GLP-1. The 1.5-hour AUC for GIP was increased with a family history of T2D [β = 1986, p = 0.004] and reduced with higher HbA1c [β = -309, p = 0.004].

It appeared that the increased 1.5-hour AUC for glucose associated with higher HbA1c was reflected differently on placebo and cafestol days. Specifically, the 1.5-hour AUC for glucose was 13% lower on the 7 mg cafestol day [797 mmol/l * min, SD 130 mmol/l * min, p = 0.05] and 6% lower on the 14 mg cafestol day [857 mmol/l * min, SD 149 mmol/l * min, p = 0.04] than on the placebo day [916 mmol/l * min, SD 127 mmol/l * min] in the upper HbA1c-tertile.

Eight participants [53%] expressed impaired glucose tolerance [IGT] [120-min glucose > 7.9 mmol/l] and/or impaired fasting glucose [IFG] [-15/0 min glucose > 6 mmol/l] on placebo day [Table 3]. In a subgroup of these participants with IGF and/or IGT, the OGTT mean curves for glucose, insulin, GLP-1 and GIP seemed to be slightly different from the subjects without IFG and/or IGT] [Figure 3]. On the 14 mg cafestol day, the subgroup’s mean 1.5-hour AUC for GIP was 11% higher [+775 pmol / l x min] than on placebo day [95%CI: 18, 1531 pmol / l x min, p=0.046]. Mean 1.5-hour AUC for glucose was 5% lower [-41 mmol / l x min] on 14 mg cafestol day than placebo day [95%CI: -100, 18 mmol / l x min, p=0.14]. Mean AUCs for insulin and GLP-1 in this subgroup were not substantially different between study days [Table 4, Figure 4]. There were no extreme outliers in AUCs in the IGF/IGT subgroup, and all AUCs were normally distributed except for 3-hour AUC for GLP-1 [Shapiro-Wilk’s test p = 0.03].

**Table 3.** Baseline data split into glucose tolerance subgroups

Impaired Glucose Tolerance and/or Fasting Glucose	NO (n=7)	YES (n=8)
**HbA1c (mmol/mol)**		
Mean	38.0	39.0
Range	35.0 - 43.0	35.0 - 47.0
SD	2.6	3.9
**Sex**		
Female	3 (43%)	1 (12%)
Male	4 (57%)	7 (88%)
**Age (years)**		
Mean	61.4	70.9
Range	20.0 - 75.0	58.0 - 76.0
SD	19.5	6.2
**Weight (kg)**		
Mean	93.3	99.9
Range	82.0 - 108.0	90.0 - 114.0
SD	11.0	7.3
**BMI - (kg/m^2^)**		
Mean	30.1	31.5
Range	27.2 - 32.1	27.2 - 34.9
SD	1.6	2.5
**Waist circumference (cm)**		
Mean	105.9	111.0
Range	92.0 - 115.0	103.0 - 116.0
SD	8.7	4.7
**HOMA-IR**		
Mean	12.7	
Range	8.2 - 19.8	9.4 - 45.8
SD	4.9	14.2
**Family history of T2D**	1 (14%)	5 (62%)

**Figure 2.**
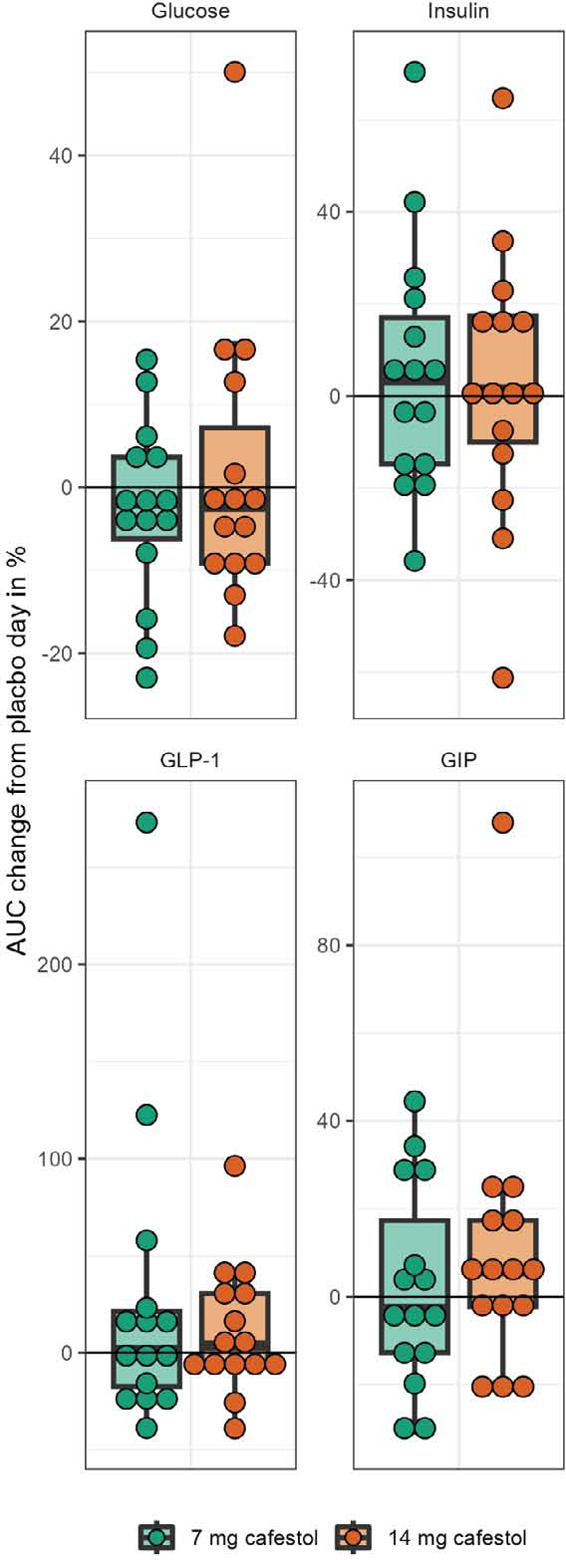
Changes in each participant's 1.5-hour area under the curve from placebo (0%) to 7 mg cafestol and 14 mg cafestol intervention, respectively. Boxes represent inter-quartile ranges. Dots represent each participant (n=15).

**Figure 3.**
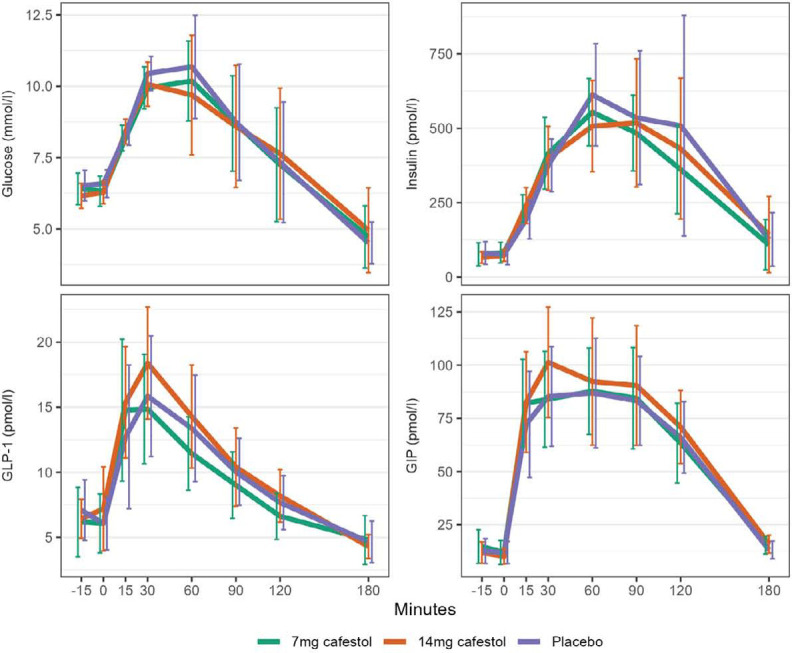
Mean curves for the impact of ingestion of 7 mg cafestol, 14 mg cafestol or placebo at time point 0 minutes on glucose, insulin, GLP-1 and GIP plasma concentrations during an oral 75 g glucose tolerance test in subgroup of participants with impaired glucose tolerance (IGT) and/or impaired fasting glucose (IFG) (n=8). Error bars represent 95% CI.

**Table 4.** 1.5- and 3-hour area under the curves in IGT/IFG-subgroup (n=8)

	Mean	SD	vs. placebo Δ-mean	T.test vs. placebo 95% CI	T.test vs. placebo p-value
**Glucose (mmol/l x min)**					
**1.5-hour AUC** Placebo	861	145			
7 mg cafestol	830	118	-31	-111, 49	0.40
14 mg cafestol	820	160	-41	-100, 18	0.14
**3-hour AUC** Placebo	1457	334			
7 mg cafestol	1428	293	-29	-210, 153	0.72
14 mg cafestol	1441	385	-16	-158, 126	0.80
**Insulin (pmol / l x min)**					
**1.5-hour AUC** Placebo	38343	14492			
7 mg cafestol	37330	10689	-1013	-11033, 9008	0.82
14 mg cafestol	36138	11656	-2205	-8952, 4542	0.47
**3-hour AUC** Placebo	73072	43209			
7 mg cafestol	64124	21537	-8948	-33406, 15511	0.42
14 mg cafestol	67625	31836	-5446	-22806, 11914	0.48
**GLP-1 (pmol / l x min)**					
**1.5-hour AUC** Placebo	1144	486			
7 mg cafestol	1080	361	-64	-314, 185	0.56
14 mg cafestol	1283	413	138	-126, 403	0.26
**3-hour AUC** Placebo	1780	697			
7 mg cafestol	1657	544	-122	-408, 163	0.35
14 mg cafestol	1936	624	156	-173, 485	0.30
**GIP (pmol / l x min)**					
**1.5-hour AUC** Placebo	6947	2793			
7 mg cafestol	7112	2478	165	-1131, 1461	0.77
14 mg cafestol	7722	3091	775	18, 1531	**0.046**
**3-hour AUC** Placebo	11565	3816			
7 mg cafestol	11694	3887	130	-1659, 1918	0.87
14 mg cafestol	12748	4503	1183	85, 2281	**0.038**

**Figure 4.**
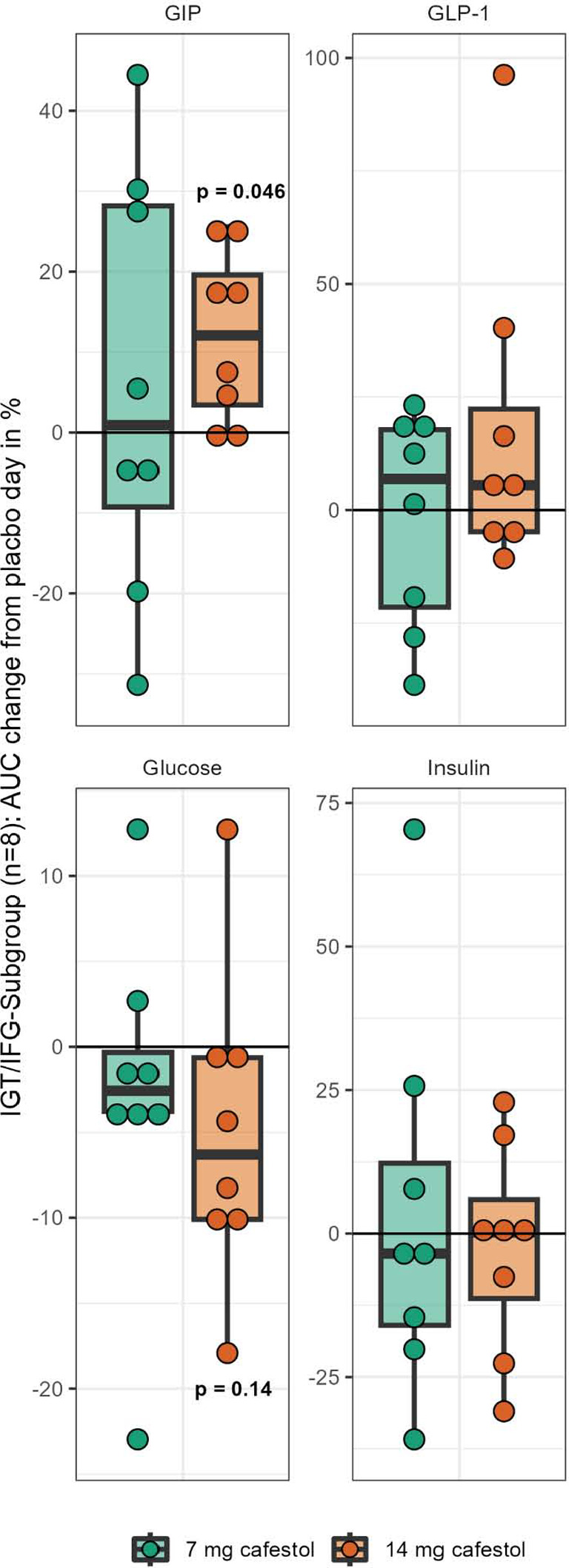
Changes in each participant's 1.5-hour area under the curve from placebo (0%) to 7 mg cafestol and 14 mg cafestol intervention, respectively. Subgroup of participants with impaired glucose tolerance (IGT) and/ or impaired fasting glucose (IFG). Boxes represent inter- quartile ranges. Dots represent each participant (n=8). p-values represent result of paired t-test with placebo within IGT/IFG subgroup.

### 3.4 Harms

No participants experienced any form of discomfort during or after study days. Four participants reported slight headaches prior to their study days during coffee consumption abstinence.

## 4 Discussion and Conclusion

While previous studies on cells and animals have shown promising results regarding the glucose level modifying effects of cafestol, its effects on glucose metabolism in humans have not yet been investigated. To our knowledge, the only randomized controlled trials performed with cafestol were conducted in the 1990’s and were mainly focused on liver enzymes and serum lipids and primarily performed using coffee oils or unfiltered coffee brews [12-15]. This study is the first to investigate cafestol’s effect on glucose metabolism in humans. The cafestol dosages were selected to mimic cafestol levels presumed achievable in a diet with normal-to-high coffee consumption [3-6 cups unfiltered coffee pr. day]. The participants experienced no discomfort after ingesting cafestol capsules. Contrary to our hypothesis, we found no acute effects of cafestol on the AUC for glucose during an OGTT in the non-diabetic subjects with abdominal obesity. Nor did we find effects of cafestol on the AUC for insulin, GLP-1 or GIP. We selected a population with abdominal obesity to study participants at risk of developing T2D; however, two thirds of the participants had HbA1c levels of 38 mmol/ mol or below and several participants showed normal glucose responses during their OGTTs. Furthermore, on placebo day, 7 participants had fasting glucose >6 mmol/l [IFG] and 3 participants had a 120-min glucose >7.9 mmol/l [IGT]. In total, only 8 [53%] unique participants had IGT and/or IGF on placebo day [Table 3]. The various conventional methods of measuring insulin resistance or glucose disposal all have pros and cons. The OGTT is a standardized straightforward way of measuring glucose disposal and to account for incretin effects; however, it is, indeed, known to render results with considerable variance. Performing this study in a more controlled and standardized setting could potentially have detected smaller effects with less noise, though perhaps in exchange for less generalizability. To sum up, we do not know if another method, e.g. the hyperinsulinemic euglycemic clamp technique or a standardized mixed meal test would have detected more prominent metabolic responses.

As already mentioned, our primary hypothesis was not supported by the study. The explanation for this can possibly be attributed to different causes. Firstly, all participants were not very insulin resistant despite having met the inclusion criterium by having a large waist circumference. Arguably, to be able to detect a cafestol-induced improvement in glucose tolerance, it may be required that participants display moderate to severe insulin resistance, and our participants may have been too glucose tolerant. Secondly, the cafestol dosage was administered concurrently with the glucose load and cafestol may not have had sufficient time to be absorbed and reach target organs before the glucose load was disposed in the body. Thirdly, cafestol may show little or no acute metabolic effects. However, contrary to this, our *in vitro* studies on human skeletal muscle cells detected an acute beneficial effect on glucose disposal and our *in vitro* studies on clonal rat beta INS-1E cells detected increased insulin secretion after acute cafestol incubation [8]. The reason for the discrepancy with the present study is unclear, and we have consequently planned both an acute cafestol- intervention study in participants with T2D, and a long- term study with sufficient time for cafestol capsules to be fully absorbed.

As previously mentioned, the effect of cafestol may have been more prominent if additional time was allowed for absorption before the glucose load was administered. Based on a study in ileostomy patients, we do expect approximately 70% of the administered cafestol to be absorbed in the small intestines [16]. The cafestol dosages [7 mg and 14 mg] administered are modest and chosen to mimic physiological circumstances experienced during traditional coffee consumption. Coffee cafestol content heavily depends on the brewing method; 100 ml of French press coffee has 2-4 mg cafestol, boiled coffee has 1-8 mg, while drip coffee has 0-0.1 mg cafestol [17]. Unfortunately, we did not have access to an analytical method to determine cafestol in the blood, which would have allowed insight into the absorption of cafestol. We discovered potentially interesting results in our exploratory ancillary analysis. Higher 1.5-hour AUC for glucose was observed in men and in individuals with higher levels of HbA1c, BMI, and age, all of which are known risk factors for T2D development. Similarly, 1.5-hour AUC for insulin was increased with BMI and reduced with workout, perhaps indicating that active and lean participants were less insulin resistant and therefore needed less insulin to metabolize the glucose load. GLP-1 was unaltered by any variable, whereas GIP was increased with family history of T2D and reduced with HbA1c. Looking only at the subgroup of participants with IFG and/or IGT on placebo day, arguably the participants with the highest risk of developing T2D, revealed meaningful nuances. Mean 1.5-hour AUC for GIP was 11% higher on 14 mg cafestol day than on placebo day [+775 pmol / l x min, p=0.046], and mean 1.5-hour AUC for glucose was 5% lower on 14 mg cafestol day than placebo day [-41 mmol / l x min, p=0.14]. Insulin and GLP-1 curves were not substantially altered. It is possible that these effects can be emphasized with a larger sample size of participants with IFG and/or IGT. Further studies in subjects with IFG and/or IGT are needed to clarify if cafestol increases GIP and reduces glucose responses to a glucose load or a mixed meal. Another subgroup, the upper HbA1c-tertile, had lower 1.5-hour AUCs for glucose on both 7 mg- [13% lower AUC, p=0.05] and 14 mg cafestol study days [6% lower AUC, p = 0.04] compared to placebo. Possible explanations for these observations could be a non-linear dose-response relationship or imprecise estimates due to large OGTT variance and a small sample size. Admittedly, with subgroups with only 5 participants in each, it is also possible that the effect of 7 mg cafestol in the upper-HbA1c-tertile was due to chance. Cafestol is known to elevate low-density lipoprotein cholesterol [LDL-cholesterol]; in a longer-term study, using an appropriate cafestol dosage is hence essential to avoid excessive LDL-cholesterol increases. Arguably, if cafestol [or even coffee] causally and dose dependently is responsible for the reduced risk of developing T2D in coffee drinkers, the repetitive nature of coffee consumption should be taken into consideration when studying cafestol’s effects. Insulin resistance, impaired glucose tolerance and T2D usually develop gradually over years. Concurrently, most coffee drinkers drink several cups of coffee every day throughout their entire lives and, intuitively, the T2D preventive effects of cafestol could manifest through habitual exposure and not acutely after ingestion.

In conclusion, we found no convincing effect of cafestol on AUC for glucose, insulin, GLP-1 or GIP in our participants with increased waist circumference. We found, however, in participants with impaired glucose tolerance and/or fasting glucose [53%], that 14 mg cafestol increased AUC for GIP and tended to lower AUC for glucose. This suggests that cafestol may contribute to coffee’s inverse association with T2D, possibly through increased GIP secretion. Our findings are novel, but further studies are needed to clarify the acute effects in participants with impaired glucose metabolism. Examining longer-term effects of cafestol ingestion on the glucose metabolism is also important, preferably in participants with impaired glucose tolerance and/or impaired fasting glucose. This may answer the question if cafestol could play a role as a future dietary supplement for prevention of T2D or adjunct treatment hereof.

## Abbreviations

T2D: Type-2 Diabetes

AUC: Area under the curve

GLP-1: Glucagon-like peptide 1

GIP: Gastric inhibitory peptide

IGT: Impaired glucose tolerance

IFG: Impaired fasting glucose

OGTT: Oral glucose tolerance test

K2EDTA Dipotassium ethylenediaminetetraacetic acid

RCF: Relative centrifugal force

ELISA: Enzyme-linked immunosorbent assay

ANOVA: Analysis of variance

BMI: Body mass index

LDL: Low-density lipoprotein cholesterol
